# Data of metal and microbial analyses from anaerobic co-digestion of organic and mineral wastes

**DOI:** 10.1016/j.dib.2019.103934

**Published:** 2019-04-19

**Authors:** Burhan Shamurad, Neil Gray, Evangelos Petropoulos, Shamas Tabraiz, Kishor Acharya, Marcos Quintela-Baluja, Paul Sallis

**Affiliations:** a School of Engineering, University of Newcastle-upon-Tyne, UK; b School of Natural and Environmental Sciences, Univesity of Newcastle-upon-Tyne, UK

## Abstract

High concentrations of minerals, heavy metals are often found in mineral wastes (MWs) originated from municipal solid waste incineration plants, so as construction/demolition sites. Such by-products (minerals) often have buffering capacity. The current work provides analysis of total and soluble (dissolved) metal concentrations released by four different MWs (a. cement-based waste, b. incineration (bottom), c. fly and d. boiler ash) supplemented to anaerobic reactors of organic waste at 37 °C. The reactors (continuous stirred tank reactor (CSTR)) were ran for 75 days at hydrolytic retention time of 20 days. Genomic DNA extraction, and qPCR and Illumina HiSeq (16S V4) analyses were conducted to investigate microbial community population and composition in anaerobic digestate samples collected from these reactors. Output data from Illumina sequencing analysis were FastQ files analysed using the QIIME2 pipeline to produce a feature table listing the frequency of each assigned microbial taxa per samples. Additional study was conducted on the microbial data to visualise variations in microbial communities using the STAMP software and phyloseq R package. Detailed interpretation and discussion of the results can be found in the related research article [1].

**Table:** Specifications table

Subject area	Chemistry, Biology
More specific subject area	Anaerobic digestion
Type of data	Text file, Table, Figure
How data was acquired	ICP-OES (Vista MPX simultaneous, USA), Illumina HiSeq (16S V4; Earlham Institute, UK)
Data format	Raw, analysed
Experimental factors	Centrifuge, filtration and acidification (for metal analysis) and centrifugation then storage at −20 °C (for molecular analysis)
Experimental features	For metal analysis, the samples were centrifuged, filtered then analysed. For molecular analysis, the samples were centrifuged genomic DNA extracted then used for microbial analyses.
Data source location	School of engineering-Newcastle University-UK
Data accessibility	Data of this manuscript is uploaded to Mendeley Data repository can be accessed through this link: https://data.mendeley.com/datasets/dkrwntknh9/draft?a=08235888-e070-4d9d-8f18-9031ded29735
Related research article	Shamurad et al. [1].Co-digestion of organic and mineral wastes for enhanced biogas production: Reactor performance and evolution of microbial community and function, Waste Manag. 87 (2019) 313–325. https://doi.org/10.1016/j.wasman.2019.02.021.

**Table:** 

**Value of the data** • Methods for metal and microbial analysis of anaerobic digestate samples are described. • Data obtained from metal analysis of mineral wastes is showing the concentration of trace elements can be amended to anaerobic reactors for enhancing anaerobic digestion (AD) processes. • The impact of the co-digestion of mineral wastes with organic wastes on the microbial community is presented. • Microbial communities observed from microbial analysis of current data can be compared with microbial communities of other AD conditions.

## 1 Data

**Fig. 1:**
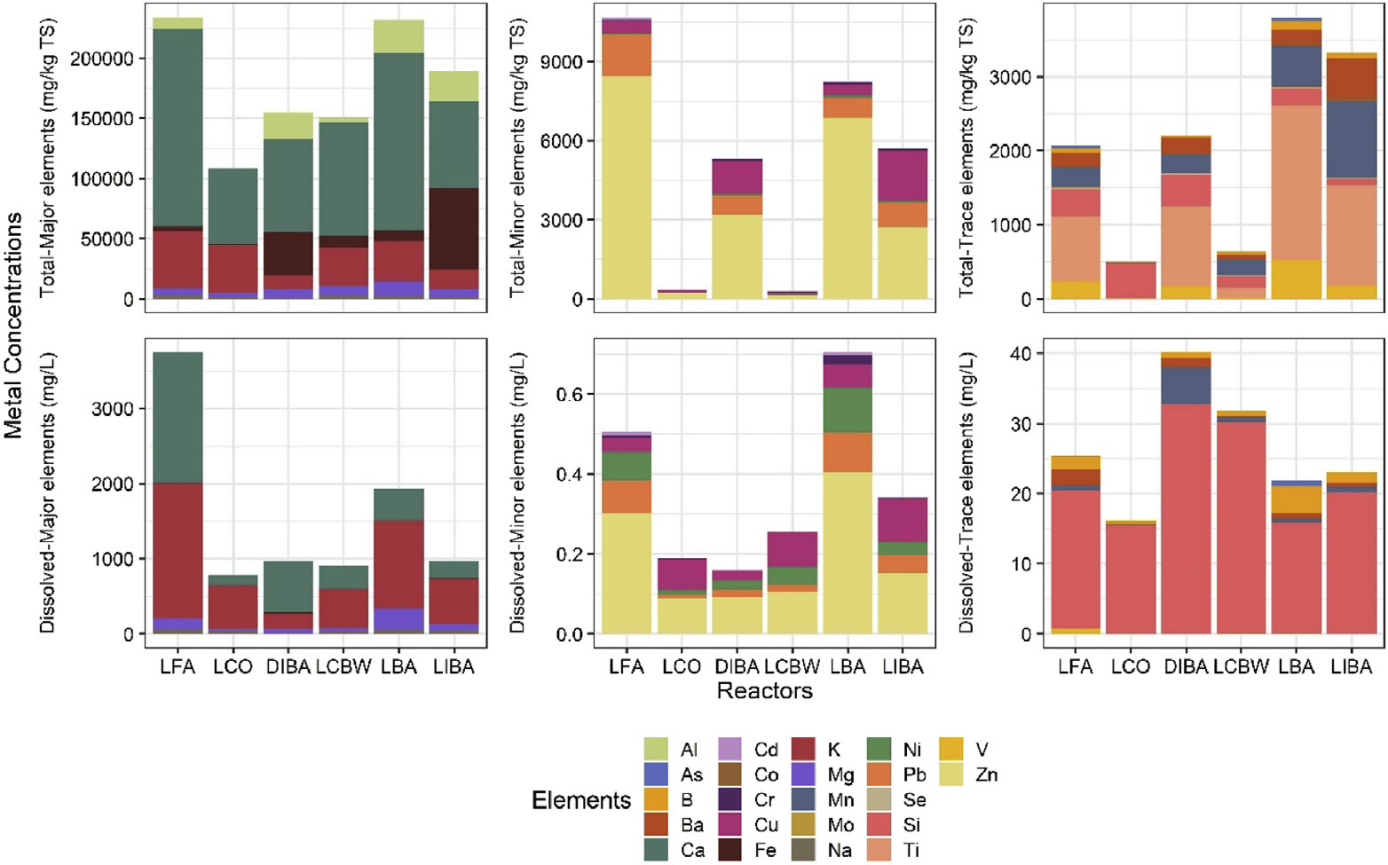
Total and dissolved metal concentrations in the reactor digestates on day 75 measured according to EPA method 3010A using ICP-OES machine. ‘Total’ is total metal concentrations, ‘Dissolved’ is dissolved (soluble) metal concentrations. CO = control reactor, IBA = incineration bottom ash, FA = fly ash, BA = boiler ash, CBW = cement-based waste. ‘L’ and ‘D’ refer to the reactors feeding method ‘liquid-recycled feeding method’ and ‘draw-and-fill feeding method’ respectively [1].

**Fig. 2:**
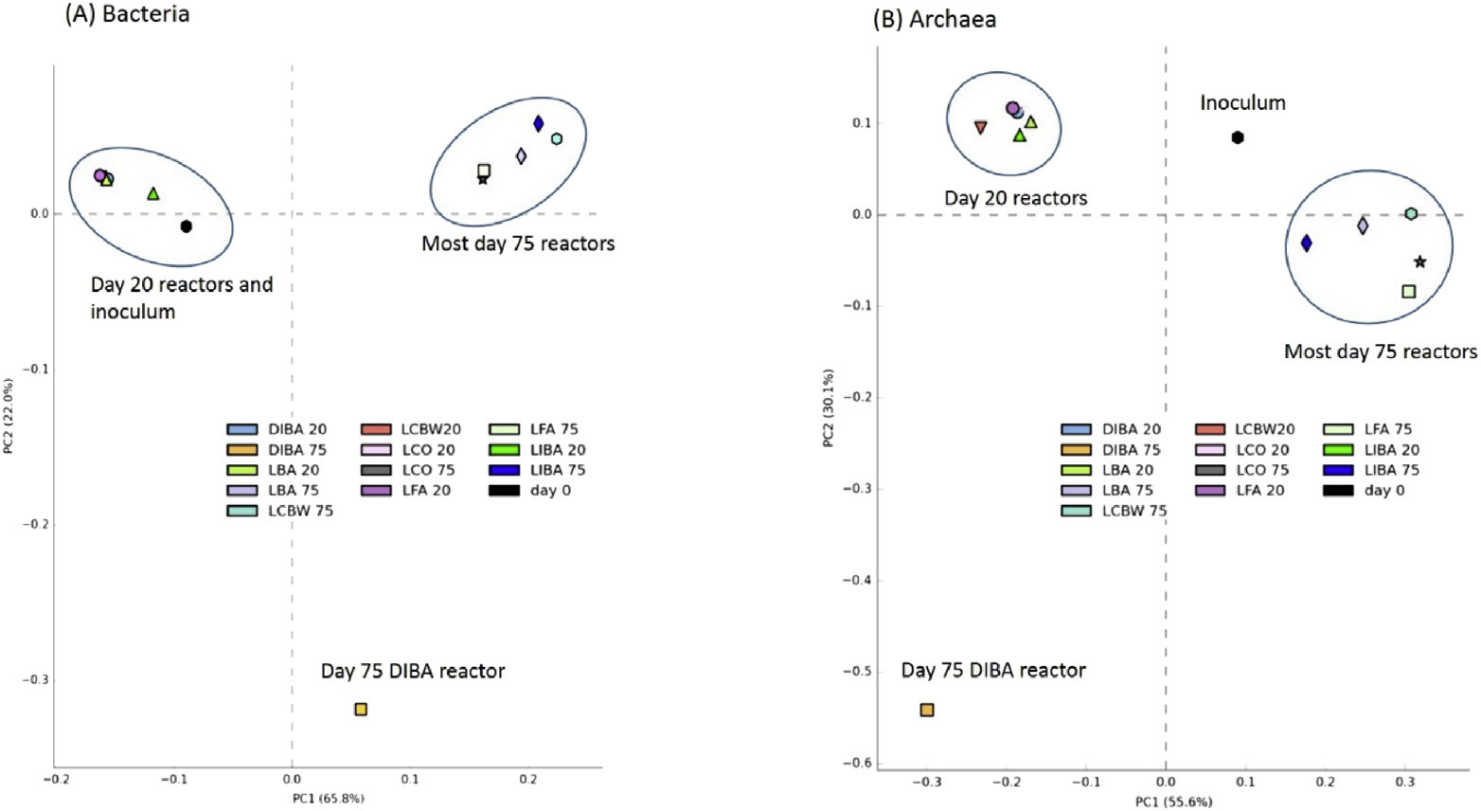
Principle component analysis of bacterial (A) and archaeal (B) communities of digestate samples collected from anaerobic reactors on days 0, 20 and 75, plots generated using STAMP software [10]. For details of the legend labels see Fig. 1.

**Fig. 3:**
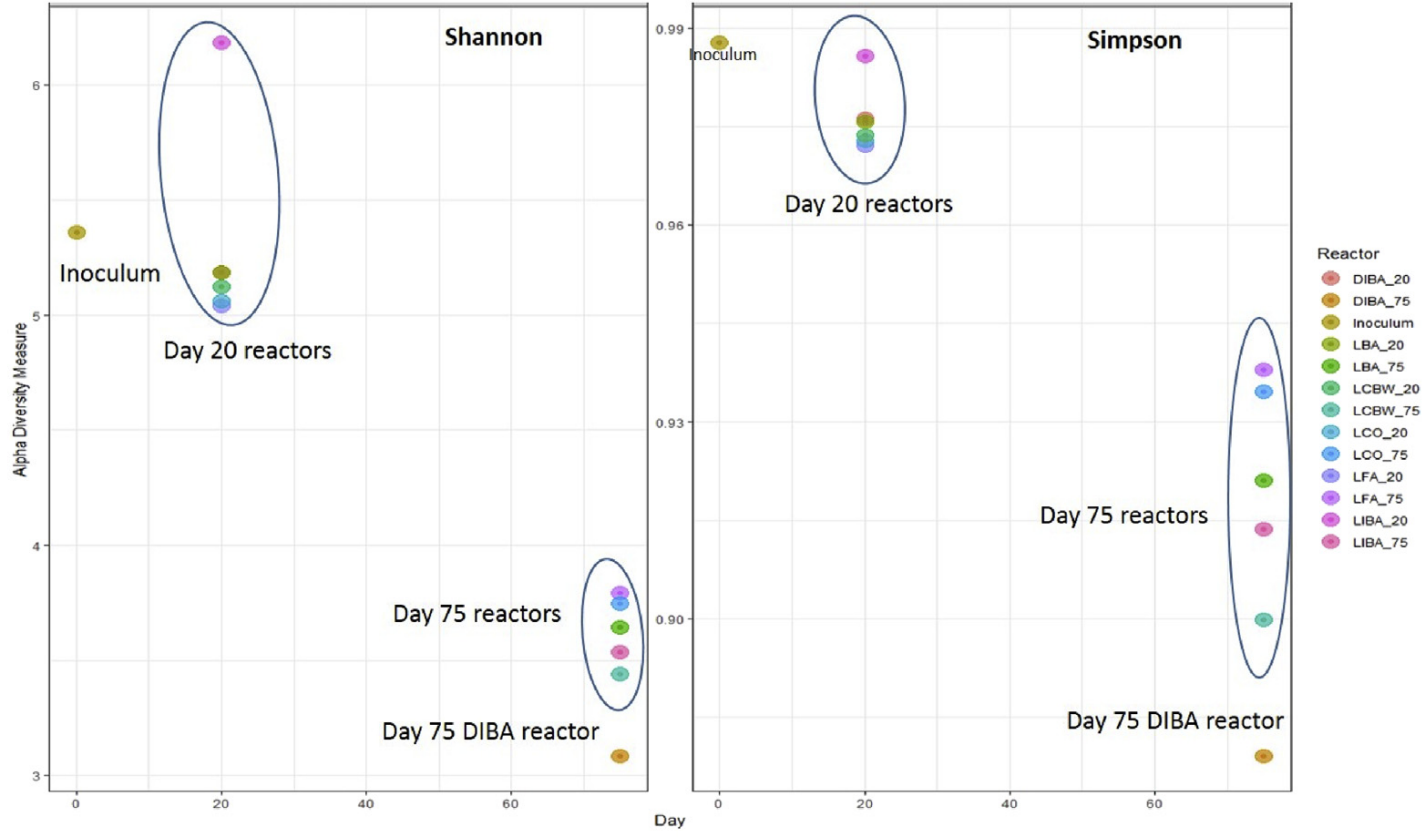
Alpha diversity metrics of microbial communities of digestate samples collected from different anaerobic reactors on days 0, 20 and 75, plots generated using phyloseq R package [11]. For details of the legend labels see Fig. 1.

**Fig. 4:**
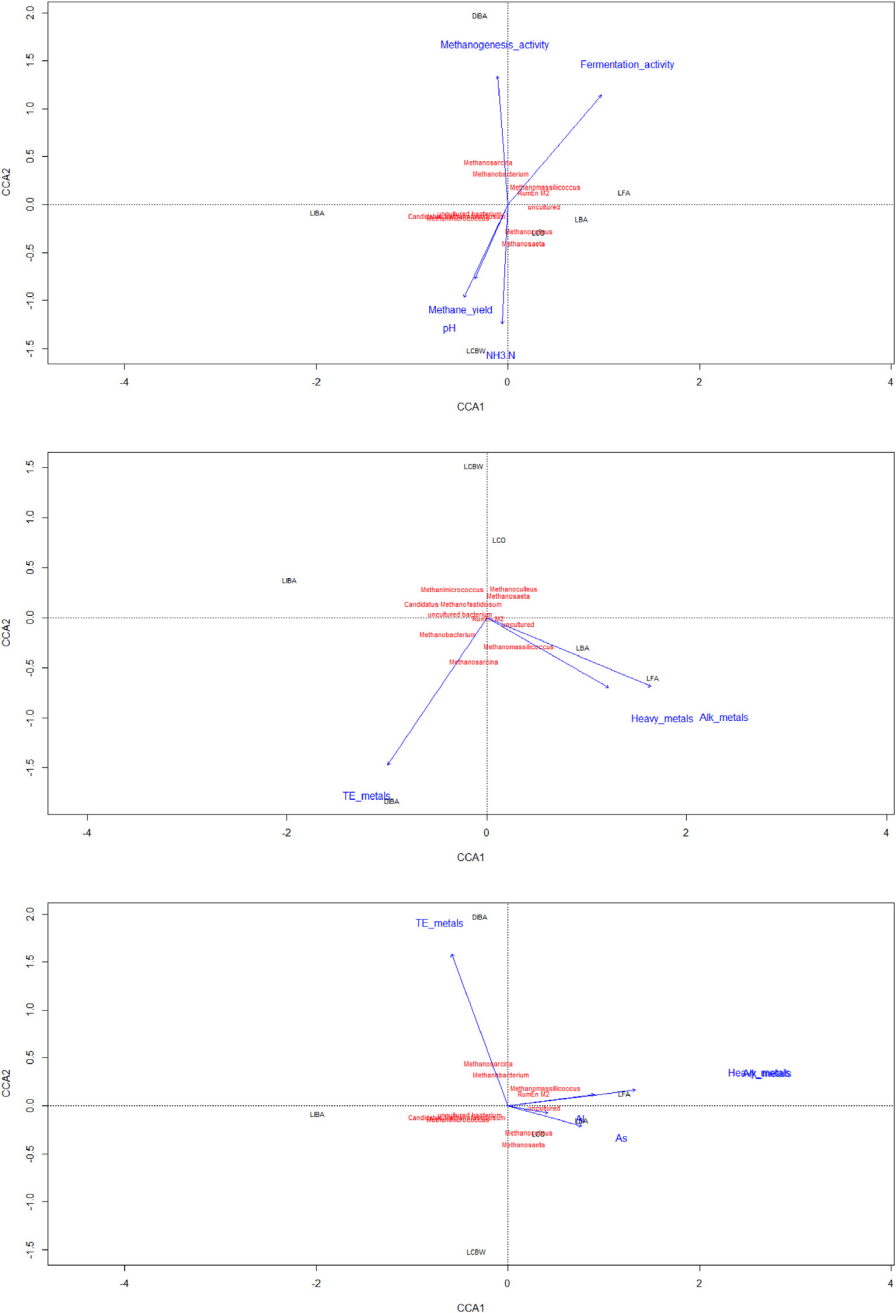
Canonical correspondence analysis (CCA) diagrams of archaeal communities (the 10 highest archaeal taxa) as determined by 16S rRNA using Vegan package [12]. Arrows indicated the direction of increasing values with the length of the arrows correlated with the percentage of community composition data accounted for by that variable. (A) Measured and calculated parameters on day 75, (B) Soluble metal concentrations combined in three groups trace (TE) metals, heavy metals and alkali (Alk) metals and (C) individual soluble metal concentrations measured [1].

The data shows metal concentrations (total and dissolved concentrations) and microbial sequencing data (Illumina HiSeq (16S V4)) of digestate samples from continuous stirred tank reactor (CSTR) systems at conventional mesophilic temperatures (37 °C) [1]. Total and dissolved concentrations of metals measured from digestate samples were classified to major, minor and trace elements (Fig. 1). The sequenced microbial communities in this data set was used for producing PCA plots (Fig. 2) showing the separation of bacterial and archaeal communities in the CSTR systems at three operation times: days 0 (inoculum), 20 and 75. Then alpha diversity of bacterial and archaeal tax were compared (Fig. 3) using Shannon and Simpson indices. Moreover, the sequencing data was used for canonical correspondence analysis (CCA; (Fig. 4)) showing correlation of the abundance of the archaeal genera with the physicochemical parameters measured/or calculated [1]. The interpretation of the metal and microbial analyses of this data set was performed by Shamurad et al. [1].

## 2 Experimental design, materials, and methods

The AD experiments were conducted in six continuous stirred tank reactor (CSTR) systems with working volume of 5L. Each reactor was a borosilicate glass quick fit flask 100 mm diameter, with three ports for feeding, sampling and mixing. The reactors were operated for 75 days. The substrate fed to each reactor was an organic waste mixed with mineral wastes (solid residues from a municipal incineration plant (solid waste based) and construction/demolition wastes). Details of reactor setup, substrate composition and operating conditions can be found in the related research article [1].

### 2.1 Determination of total and soluble metal concentrations

#### 2.1.1 Preparing digestate samples for total and soluble metal concentrations

Total concentrations of metals in anaerobic digestate samples of mesophilic reactors (operated for 75 days) were measured according to the EPA method 3010A. Representative mass (2 g) of dried (at 50 - 70 °C) and crushed digestate samples were measured then transferred to long digestion glass tubes prior to acidic digestion with concentrated HNO_3_ and HCl at room temperature for 16 hours, and then boiled on a heating block for another 1 h at 100 °C. After cooling, the acid digested samples were filtered through acid resistant filter papers (Whatman ash-less filter papers) then diluted using 0.5 M HNO_3_. The diluted samples were analysed for total metal concentrations using ICP—OES (Vista MPX simultaneous ICP-OES).

Soluble (dissolved) metal concentrations in anaerobic digestate samples were measured from supernatant solutions discarded from digestate samples centrifuged for 30 min at 3392 × *g* (Sigma centrifuge, UK). The samples were acidified with concentrated HNO_3_ (1 - 2 drops per sample) then diluted with 0.5 M HNO_3_ and stored at 5 °C until analysed by ICP-OES.

#### 2.1.2 Elemental analysis by ICP-OES

Concentrations of the elements in digestate samples (section 2.1.1) were quantified by ICP-OES, which uses emission spectra of a sample to identify the elements and measure their concentrations. A main calibration standard solution (Standard-1; Table 1) was prepared from stock solutions (stock solution of each element was 1000 ppm concentration) of the measured elements. Then another two calibration standard solutions were prepared by diluting the main calibration standard solution (dilution factors were 1/10 and 1/100) to construct multipoint standard curves covering the range of element concentrations anticipated in the samples. The 0.5 M HNO_3_ solution used for diluting the acid digested samples (section 2.1.1) was used as a matrix solution for preparing the calibration standard solutions.

**Table 1:** Concentration of elements in the standard solutions used for constructing calibration curves of the ICP-OES machine.

Concentration of elements (ppm)			
Elements	Standard-1	Standard-2	Standard-3
Ca	150	15	1.5
Mg	15	1.5	0.15
Na	15	1.5	0.15
K	150	15	1.5
Fe	50	5	0.5
Mn	50	5	0.5
Al	150	15	1.5
Si	50	5	0.5
Cd	5	0.5	0.05
Cr	10	1	0.1
Co	1	0.1	0.01
Cu	10	1	0.1
Ni	5	0.5	0.05
Pb	25	2.5	0.25
Ti	50	5	0.5
V	3	0.3	0.03
As	2	0.2	0.02
B	2	0.2	0.02
Ba	50	5	0.5
Se	2	0.2	0.02
Mo	5	0.5	0.05
Zn	5	0.5	0.05

#### 2.1.3 Quality control

In order to ensure the absence of sample contamination, blank and standard samples were prepared following the same sample-preparation and analytical processes. Replicate samples were processed, and the accuracy of the ICP-OES machine was determined by running the blank and standard samples after every 10 samples analysed.

### 2.2 Microbiology analyses

#### 2.2.1 DNA extraction

Total genomic DNA of biomass samples (obtained after centrifugation (5 min, 15.000×*g*) of 1 mL of each digestate sample) were extracted according to the method described in Ref. [2]. The absence/or presence of PCR inhibitors in the DNA was evaluated using a Nanodrop (Thermo Fisher, UK). The acceptable range between 1.8 and 2.2 was ensured for the DNA quality ratios of 260:280 and 230:260. For quality control, with each batch of DNA extraction, blank DNA samples were prepared following the same sample-preparation and DNA extraction methods. The blank samples were analysed with each batch of Real-time PCR and Illumina sequencing analyses.

#### 2.2.2 Real-time PCR (qPCR) standards

The mcrA gene was targeted to measure the abundance of methanogens (archaea) in the digestate samples. *Methanosarcina barkeri* pure cultures were used to prepare the mcrA gene standard for qPCR analysis. The DNA extraction was carried out using an MP-bio ‘for soil DNA’ extraction kit (UK) based on the manufacturer's directions. The amplification of the mcrA gene was carried out using the mlas-F primer as per Steinberg et al., 2009 [3]. The generated PCR products were employed for the mutation of *Escherichia coli* cells as per the manufacturer of the kit used (TA cloning kit; Invitrogen, UK). The clones that were found mutated (positives) were then incubated at 37 °C using LB broth as growth medium (spiked with ampicillin). The generated plasmids were then extracted and cleaned (purification) using a purification kit for plasmids (ROCHE, UK). The yields were then quantified using Quant-It (Invitrogen, UK). Quantification enabled dilution (using PCR-grade distilled water) of the plasmid DNA from each clone to generate serial dilutions with known populations (ranged between 10^2^ to 10^8^ gene copies/μL). These populations were used for the qPCR standards.

For measuring bacteria abundance in the digestate samples, the 16S rRNA gene was targeted. To prepare the 16S rRNA standard for qPCR analysis, the complete 16S rRNA gene was amplified from *E. coli* using the PA/PH primers (pA and pH primers [4]; Table 2)). PCR reaction was conducted using Phusion Flash High-fidelity PCR master mix (ThermoFisher), using the following thermocycle program: (i) 10 sec denaturation @ (98 °C), (ii) 35 cycles of 1 sec denaturation (98 °C), (iii) 5 sec annealing (98 °C), (iv) 15 sec elongation (72 °C), and (v) 1 min elongation (72 °C). The products were separated on 1.5% agarose gel electrophoresis containing SYBR^®^ safe DNA gel stain (Sigma) and visualized using GelDoc (Biorad). The generated PCR products were then purified using the GenElute PCR clean-up kit (Sigma-Aldrich) as per the manufacturer's instructions. The TOPO pCR4 vector (Invitrogen) kit was used for the cloning of the purified products. the fresh cloned plasmids were re-purified with the PureYield Plasmid Miniprep System (Promega). The Quant-iT Picogreen dsDNA Assay kit (Invitrogen, Life Technologies, Inc.) with the SpectraMax^®^ M3The plasmid was used for the quantification of the DNA concentration. The absolute number of the gene copies of the genes used for the standards was calculated based on the plasmid size and insert length (3973 and 1515 bp respectively) assuming a mass of 660 Da/bp (molecule). The initial stock solution of the reference DNA used for the generation of the qPCR standards contained 10^9^ gene copies/μL.

**Table 2:** Primer design of the qPCR analysis targeting 16S rRNA gene.

Target	Primer	Sequence (5′ – 3′)	Product size (bp)	Tm	Reference
16S rRNA	pA	AGAGTTTGATCCTGGCTCAG	1515	55	[4]
	pH	AAGGAGGTGATCCAGCCGCA			
16S rRNA	1055F	ATGGCTGTOGTCAGCT	337	60	[5]
	1392R	ACGGGCGGTGTGTAC			

#### 2.2.3 qPCR analysis

For the quantification of the methanogenic and bacterial population in the bio-reactor (digester) Real-time PCR analysis (qPCR) was used. The methanogenic population was quantified followed the protocol above as well as the protocol provided by Steinberg et al., 2009 [3]. For the qPCR a CFX96 real–time PCR system (Biorad, UK) was used. The conditions set for the reaction (mcrA gene) included: (i) 3 min initial denaturation at 98 °C, (ii) 39 cycles of denaturation at 95 °C for 5 sec, (iii) annealing at 66 °C for 10 sec, (iv) extension at 65 °C for 5 sec with a 0.5 °C increment, and (v) final extension step at 95 °C for 0.5 min as per the manufacturer's protocol (BIORAD, UK for Ssofast Evergreen^®^ Supermix). The reaction solution contained 1 μL sterile de-ionized water, 3 μL of sample DNA template, 0.5 μL each of the forward and reverse primers (the primers were diluted to concentration of 10 pmol/μL) and 5 μL of Ssofast EvaGreen Supermix solution (Biorad, UK). The analysis was carried out based on a 5-point calibration curve using the mcrA gene standards that were prepared followed the protocols above. For the dilutions filter sterile de-ionized water was used. All qPCR reactions were performed in triplicates; the reactions' efficiency was estimated based on the curve generated by the standards. This was automatically assessed by the instrument's software.

Total bacteria was quantified using a SYBR green-based method assay (forward (1055F) and reverse (1392R) primers [5]; (Table 2)). SYBR-green reactions were conducted using SsoAdvanced™ Universal SYBR^®^ Green Supermix (BioRad) as reagent. The reaction followed a thermocycle program with: (i) 2 min of initial denaturation (98 °C), (ii) 40 cycles of 5 sec denaturation (98 °C), and (iii) 5 sec annealing/extension (60 °C). All assays were carried out in triplicates using a BioRad CFX C1000 System (BioRad, Hercules, CA USA). To avoid inhibition phenomena during the amplification the DNA samples were diluted to a working solution of 5 ng/μL. An internal control DNA was also employed in the SYBR-green reactions to assure there is no errors in the quantification process related to contamination. A standard curve with known copy numbers (10^3^ and 10^8^) was incorporated using plasmid clones of target sequences (more details given in section 2.2.2). The reactions were all carried out in triplicates. For enumeration of the 16S rRNA gene via qPCR the following mixture was prepared: 3 μL template DNA, 5 μL Ssofast EvaGreen Supermix (Bio-Rad, UK), 0.5 μL of forward (1055F) and reverse (1392R) primers [5]; (Table 2.), and 1 μL sterile de-ionized water (total volume of 10 μL).

Detailed information on the abundance of bacteria and methanogens (archaea) from qPCR analysis can be seen in the related research article [1]. One-way ANOVA analysis was conducted in SPSS (IBM SPSS Statistics version 23) for the measured physicochemical parameters (such as soluble COD (sCOD), pH and NH_3_—N) of digestate samples and the calculated hydrolysis activity and methanogenesis activity values of reactors on day 75. Table 3 shows results of one - way ANOVA analysis.

**Table 3:** Results of one-way ANOVA analysis for measured and calculated parameters on day 75 of mesophilic anaerobic digestion of organic and mineral wastes [1].

		Sum of Squares	Mean Square	F	Significant
Methane Yield (mL CH_4_/g VS added)	Between Groups	20332	20332	1.222	0.331
	Within Groups	66553	16638		
	Total	86885			
Hydrolysis activity (pgram COD/cell/d)	Between Groups	0.002	0.002	2.425	0.194
	Within Groups	0.003	0.001		
	Total	0.005			
Methanogenesis activity (pmol CH_4_/cell/d)	Between Groups	0.097	0.097	428.831	0
	Within Groups	0.001	0		
	Total	0.098			
Soluble COD (sCOD) (mg/L)	Between Groups	24999	24999	0.051	0.832
	Within Groups	1949055	487264		
	Total	1974053			
pH	Between Groups	0.645	0.645	2.667	0.178
	Within Groups	0.968	0.242		
	Total	1.613			
NH_3_N (mg/L)	Between Groups	120460	120460	85.33	0.001
	Within Groups	5647	1412		
	Total	126107			

#### 2.2.4 Illumina sequencing of 16S rRNA gene

The sequencing data was obtained from a 16S rRNA library (Illumina HiSeq (16S V4)) prepared by Earlham Institute (UK) after samples’ quantification (Qubit dsDNA HS Assay Kit (Thermo Fisher Scientific Q33231)) and purification. The purity was inspected using the Drop Sense 96 (PerkinElmer).

Prior PCR amplification the DNA samples were diluted to reach a mass of 10 ng. The amplification (PCR) was carried out using 2 μL of the forward and reverse primers (each) [6] at a concentration of 2.5 μM; 0.1 μL of Kapa 2G Robust polymerase (Kapa Bio systems KK5005), 0.5 μL 10 mM dNTPs were also added. Finally, Qiagen nuclease free water (Qiagen 129114) was added to make a volume of 25 μL. The amplification program had (i) 3 min of initial denaturation at 94 °C, and 25 cycles of (ii) denaturation at 94 °C for 45 sec, 55 °C for 15 sec, and 72 °C for 30 sec, then (iii) final extension for 3 min at 72 °C, and (iv) holding for 3 min at 4 °C. All amplified DNA samples were then purified using the Agencourt AMPure XP bead clean-up kit (Beckman Coulter A63882) using the manufacturer's protocol modified by two 80% EtOH washes and re-suspension of the samples in 25 μL of elution buffer (10 mM Tris). The generated libraries were quantified (Qubit dsDNA HS Assay Kit) and sized via PerkinElmer GX using a highly sensitive DNA chip (PerkinElmer CLS760672). Afterwards, all libraries were equimolar-pooled; the pool was quantified via qPCR using a Kapa Library Quantification Kit (Kapa Biosystems KK4828). The sequencing data deposited in Mendeley Data repository (https://data.mendeley.com/datasets/dkrwntknh9/draft?a=08235888-e070-4d9d-8f18-9031ded29735).

#### 2.2.5 Sequenced data processing

The FastQ files of the sequenced data were de-multiplexed and quality filtered in DADA2 [7], closed reference operational taxonomic unit (OTU) picking was performed using VSEARCH [9] within the QIIME2 pipeline (https://qiime2.org, [8]). In QIIME2 a table of representative sequences in the samples was produced. Then the sequences in this table were compared with those available in the SILVA119 reference database to produce a feature table containing the frequencies of each taxon per samples. For representative visualization of the data produced and to highlight the differences in the microbial community structure further analysis was carried out (figures). Plots of principle component analysis (PCA) of bacterial and archaeal (Fig. 2) communities and of alpha diversity metrics (Fig. 3) from different AD reactors and time points were generated using STAMP software [10] and phyloseq R package [11]. Moreover conical correspondence analysis (CCA) was conducted (Fig. 4) to relate community composition to measured (soluble metal concentrations) and calculated parameters (hydrolysis and methanogenesis activity) on day 75 using Vegan R package [12].

## References

[1] Shamurad B., Gray N., Petropoulos E., Tabraiz S., Acharya K., Quintela-Baluja M., Sallis P. (2019). Co-digestion of organic and mineral wastes for enhanced biogas production: reactor performance and evolution of microbial community and function. Waste Manag..

[2] Griffiths R.I., Whiteley A.S., O'Donnell A.G., Bailey M.J. (2000). Rapid method for coextraction of DNA and RNA from natural environments for analysis of ribosomal DNA- and rRNA-based microbial community composition. Appl. Environ. Microbiol..

[3] Steinberg L.M., Regan J.M. (2009). mcrA-targeted real-time quantitative PCR method to examine methanogen communities. Appl. Environ. Microbiol..

[4] Edwards U., Rogall T., Blöcker H., Emde M., Böttger E.C. (1989). Isolation and direct complete nucleotide determination of entire genes. Characterization of a gene coding for 16S ribosomal RNA. Nucleic Acids Res..

[5] Harms G., Layton A.C., Dionisi H.M., Gregory I.R., Garrett V.M., Hawkins S.A., Robinson K.G., Sayler G.S. (2003). Real-time PCR quantification of nitrifying bacteria in a municipal wastewater treatment plant. Environ. Sci. Technol..

[6] Kozich J.J., Westcott S.L., Baxter N.T., Highlander S.K., Schloss P.D. (2013). Development of a dual-index sequencing strategy and curation pipeline for analyzing amplicon sequence data on the MiSeq Illumina sequencing platform. Appl. Environ. Microbiol..

[7] Callahan B.J., McMurdie P.J., Rosen M.J., Han A.W., Johnson A.J.A., Holmes S.P. (2016). DADA2: high-resolution sample inference from Illumina amplicon data. Nat. Methods.

[8] Caporaso J.G., Kuczynski J., Stombaugh J., Bittinger K., Bushman F.D., Costello E.K., Fierer N., Peña A.G., Goodrich J.K., Gordon J.I., Huttley G.A., Kelley S.T., Knights D., Koenig J.E., Ley R.E., Lozupone C.A., McDonald D., Muegge B.D., Pirrung M., Reeder J., Sevinsky J.R., Turnbaugh P.J., Walters W.A., Widmann J., Yatsunenko T., Zaneveld J., Knight R. (2010). QIIME allows analysis of high-throughput community sequencing data. Nat. Methods.

[9] Rognes T., Flouri T., Nichols B., Quince C., Mahé F. (2016). VSEARCH: a versatile open source tool for metagenomics. PeerJ.

[10] Parks D.H., Tyson G.W., Hugenholtz P., Beiko R.G. (2014). STAMP: statistical analysis of taxonomic and functional profiles. Bioinformatics.

[11] McMurdie P.J., Holmes S. (2013). Phyloseq: an R package for reproducible interactive analysis and graphics of microbiome census data. PLoS One.

[12] Dixon P. (2003). VEGAN, a package of R functions for community ecology. J. Veg. Sci..

