# Supplementary material for: Data of metal and microbial analyses from anaerobic co-digestion of organic and mineral wastes
Source: Data Brief. 2019 Apr 19;24:103934. doi: 10.1016/j.dib.2019.103934 (PMC6502732; doi:10.1016/j.dib.2019.103934)
Supplement: Supplementary file 1 — Multimedia component 1 [file mmc1.pdf]

Declaration on conflict of interest

We declare that there is no conflict of interest.
